# Editorial: Polymer materials for energy storage and harvesting, and other sustainable applications

**DOI:** 10.3389/fchem.2023.1352000

**Published:** 2023-12-19

**Authors:** Matteo Bonomo, Zhuoxin Liu, Alessandro Piovano, Zifeng Wang

**Affiliations:** ^1^ Department of Chemistry and NIS Interdepartmental Center and INSTM Reference Centre, University of Turin, Turin, Italy; ^2^ National Reference Center for Electrochemical Energy Storage (GISEL)—INSTM, Firenze, Italy; ^3^ College of Materials Science and Engineering, Shenzhen University, Shenzhen, China; ^4^ GAME Lab, Department of Applied Science and Technology, Polytechnic of Torino, Torino, Italy; ^5^ School of Health Science and Engineering, University of Shanghai for Science and Technology, Shanghai, China

**Keywords:** nanocomposites, energy storage, energy conversion, sustainability, biopolymers, CO2 capture, waste management

Polymer materials, together with their composites, are at the center of the scientific discussion for the development of a more sustainable society. They play an important role in many technological applications, while bringing critical issues for the environment after being consumed. As a matter of fact, plastics hold the potential to provide versatile solutions for the challenges encountered in the fields of energy harvesting and storage (especially for what concerns the booming of flexible electronics, requiring adequate power supply), filtering and adsorbing systems (with high specific surface area and tunable porosity), and food packaging (lightweight, mechanically resistant, and chemically inert).

The Research Topic aimed at pointing out the crucial role of polymers in sustainability, covering the whole field from the synthesis of new materials to their employment into practical devices, inspiring further research and paving the way to new discoveries and developments.

For instance, an innovative frontier in the use of polymeric compounds in energy storage devices (i.e., application in electrochromic energy storage devices) has been clearly summarized by Liu et al. Recent advances, reported there in, highlight a promising role of well-established polymers such as PPy (polypirrole) or PANI (polyaniline) in electrochromic battery. As a paradigmatic example, PANI (even *in-situ* polymerized) have been proposed as electrochromic electrode for both monovalent (*i.e.*, Li^+^) or polyvalent (*i.e.*, Zn^2+^ or Al^3+^) metal-ion batteries, showing very promising capacity and stability longer than 1,000 cycles. Beside batteries, electrochromic polymers could also be exploited as electrodes in flexible supercapacitors delivering specific capacitance exceeding 300 F g^−1^ and withstanding bending and stretching cycles. The last section of this mini review is devoted to the description of electrochromic solar cells. Albeit, the efficiency of this devices is still far from the state-of-the-art, the exploitation of electrochromic ability of the polymeric active layers would be very interesting for Building Integrated Photovoltaics projects.

In order to pursuit sustainable environmental solutions, not only energy storage is at the forefront of scientific research, but also carbon dioxide capture. These challenges are crucial in the context of escalating climate change concerns and the growing need for renewable energy sources. In this landscape, Alenezi et al. intricately ties into the pressing need for innovative materials capable of mitigating environmental impact. The authors have successfully synthesized nitrogen-enriched, KOH-activated porous carbons from polycarbazole phthalonitrile networks. This material not only addresses the crucial issue of CO_2_ capture by achieving an impressive uptake of 19.5 wt% at 0°C but also demonstrates good energy storage capabilities, making it a potential material for supercapacitor electrodes. With a specific surface area of 1,279 m^2^ g^−1^ and an energy storage capacity of up to 451 F g^−1^, along with robust durability (retaining 95.9% capacity after 2000 cycles), this material stands out as a dual-functional solution that is both efficient and sustainable. This study not only contributes to the field by offering a viable material for CO_2_ adsorption but also highlights the potential for multifunctional applications in energy storage.

Recently, the development of new synthetic materials goes hand in hand with the readjustment of natural ones. This is the case, for example, of natural biopolymers, which are obtained from various sources, including plants (e.g., rice, cotton, and rubber), animals (e.g., skin, meat, and bones), microbes (bacteria and virus), and even agriculture waste, and differ from their petroleum-derived counterparts in terms of physiochemical properties, biological or environmental friendliness. Wypij et al. summarized the recent strategic applications of natural polymer-based nanocomposites in the form of nanocarriers, polymer hydrogels, nanocoatings, and nanofilms for food and agriculture. As multiphase hybrids, polymer nanocomposites consist of polymer matrix, nanofibers, plasticizers, and other additives. Natural biopolymers and degradable synthetic polymers have been attracting increasing research attention due to the discovery of adverse effects of micro-nanosized plastics on the environment, ecological systems, and health. Due to fast degradability, natural biopolymers and composites could be decomposed into nontoxic small molecules by microorganisms. There are many modern techniques to characterize the properties of biopolymers, including chemical bonding information, crystallinities, composition, morphological information, etc. Therefore, complete properties sketch of the biopolymers could be potentially obtained. By using eco-friendly biopolymers, agrochemicals (as nanofertilizers, micronutrients, and pesticides) could be delivered to the soil and water to improve the agricultural productivity. This could be achieved by introducing the targeted chemicals into the cavity of the nanocarriers. As for coatings, the biopolymers exhibited advantages in preparing edible or nano-active packaging for fruits in replacement of waxes. Such functional coatings made of lipids, polysaccharides, or proteins should obtain barrier capabilities to oxygen gas and water vapor. Despite the tremendous progress made, the risk of using nano-biopolymers should be carefully evaluated by various standardized testing and controlling methods.

Despite the remarkable contribution of plastics to the development of the society, the disposal of postconsumer plastic waste is increasingly more demanding. Nowadays, the mostly adopted strategy is incineration, which allows energy recovery but arises many concerns due to the emission of greenhouse gases and the depletion of essential and limited resources obtained from petroleum. Shah et al. propose pyrolysis and gasification as alternative routes for the future, describing in detail the two processes and comprehensively reviewing the main results obtained. Generally speaking, pyrolysis and gasification that convert organic substances into liquid and gas fuels, together with tar, coke and char. In particular, pyrolysis is a thermal decomposition under inert atmosphere, while the gasification implies a subsequent partial oxidation by reaction with a gas stream (usually air, oxygen, or steam) to obtain mostly syngas. The variable composition and quality of the plastics feed is a challenge for both processes, whose economic feasibility relies on the possibility of treating mixed plastics waste without any sorting step, converting it into a mixture of light hydrocarbons suitable for petrochemical refineries. The authors analyze the parameters that can be adopted to control the two processes, with particular focus at the temperature and the heating rate to control the distribution of the products. Overall, the paper constitutes a guideline for further development of these two processes, so to close the loop of polymer materials.

The influence of plastic materials on sustainability is a huge and continuously evolving topic that cannot be exhaustively covered by whatever articles Research Topic, but the snapshots included in this Research Topic serve to raise the awareness of this too often mistreated nexus and to inspire further researches by the new generations of scientists.

